# Prevalence and types of errors in the electronic health record: protocol for a mixed systematic review

**DOI:** 10.1136/bmjopen-2024-098241

**Published:** 2025-06-09

**Authors:** Anna Kharko, Maria Hägglund, Dafina Angelova, Therese Scott Duncan, Josefin Hagström, Kirralise Hansford, Joanne Hunt, Ylva Trolle Lagerros, Cosima Locher, Brian McMillan, Asha-Judy Nivins, Sophie Anna Rosch, Julian Schwarz, Saija Simola, Charlotte Blease

**Affiliations:** 1 Department of Women and Children’s Health, Uppsala University, Uppsala, Sweden; 2 Centre for Primary Care and Health Services Research, The University of Manchester, Manchester, UK; 3 MedTech Science & Innovation Centre, Uppsala University Hospital, Uppsala, Sweden; 4 Department of Obstetrics and Gynaecology, NIHR Cambridge Biomedical Research Centre, Cambridge, UK; 5 Department of Physiology, Development, and Neuroscience, Cambridge University, Cambridge, UK; 6 Nuffield Department of Women’s and Reproductive Health, University of Oxford, Oxford, UK; 7 Department of Endocrinology, Metabolism and Diabetes, Karolinska University Hospital, Stockholm, Sweden; 8 Department of Consultation-Liaison Psychiatry and Psychosomatic Medicine, University Hospital Zurich, Zürich, Switzerland; 9 Faculty of Psychology, University of Basel, Basel, Switzerland; 10 Immanuel Klinik Rüdersdorf, Rudersdorf, Germany; 11 Department of Computer Science, Aalto University, Aalto, Finland; 12 Digital Psychiatry, Beth Israel Deaconess Medical Center, Boston, Massachusetts, USA

**Keywords:** eHealth, Electronic Health Records, GENERAL MEDICINE (see Internal Medicine), Health & safety, Systematic Review, Delivery of Health Care, Integrated

## Abstract

**Abstract:**

**Introduction:**

In countries with access to the electronic health record (EHR), both patients and healthcare professionals have reported finding errors in the EHR, so-called EHRrors. These can range from simple typos to more serious cases of missing or incorrect health information. Despite their potential detrimental effect, the evidence on EHRrors has not been systematically analysed. It is unknown how common EHRrors are or how they impact patients and healthcare professionals.

**Methods and analysis:**

A mixed systematic review will be carried out to address the research gap. We will search PubMed, Web of Science and CINAHL for studies published since 2000, which report original research data on patient-identified and healthcare professional-identified EHRrors. We will analyse (1) the prevalence of EHRrors, (2) the types of EHRrors and (3) their impact on care. Quantitative and qualitative findings will be synthesised following the Joanna Briggs Institute Framework for Mixed Systematic Reviews. Identified studies will be critically appraised for meta-biases and risk of bias in individual studies. The confidence in the emerging evidence will be further assessed through the Grading of Recommendations Assessment, Development and Evaluation approach. Findings will be contextualised and interpreted involving an international team of patient representatives and practising healthcare professionals.

**Ethics and dissemination:**

The study will not involve collection or analysis of individual patient data; thus, ethical approval is not required. Results will be published in a peer-reviewed publication and further disseminated through scientific events and educational materials.

**PROSPERO registration number:**

CRD42024622849.

**STRENGTHS AND LIMITATIONS OF THIS STUDY**

- The prevalence of errors in electronic health records (EHRrors) remains poorly understood, in part due to a fragmented body of literature. This review will systematically analyse the existing evidence, focusing on the perspectives of the most impacted stakeholders: patients and healthcare professionals.
- A rigorous approach will be employed using the Joanna Briggs Institute Framework for Mixed Systematic Reviews, Mixed Methods Appraisal Tool for quality appraisal and Confidence in the Evidence from Reviews of Qualitative Research to assess confidence in findings, ensuring comprehensive synthesis of mixed-methods evidence on EHRrors and their impact.
- Findings will be reported in strict compliance with the Preferred Reporting Items for Systematic Review and Meta-analysis Protocols guidelines, ensuring transparency and replicability.
- The search will be conducted exclusively in English, which may omit relevant research with abstract and title in other languages.

## Introduction

 The digitisation of health records has done more than simply move patient history from paper to online. Electronic health records (EHRs) have transformed documentation and delivery of care. Introduced as a measure to counter decentralised fragmented record keeping, EHRs promise better care coordination^1^ and higher quality of care.^2^ The transition, however, has been complex, bringing both improvements and new challenges.

Compared with paper records, EHRs enable healthcare professionals (HCPs) to produce more legible and comprehensive patient records.^3^ Patient data are maintained in standardised and reusable formats that further allow clinical decision support systems.^4^ Yet, EHRs also impose new burdens. HCPs report an increase in administrative duties due to spending more time on documentation,^5^ including outside working hours.^6^ The extended workload associated with EHR use has been linked to burnout^7 8^ and reduced job satisfaction.^9^ In parallel, EHRs have continued to change.

A significant development has been the introduction of patient access to the EHR. Today, nearly 30 countries have opened the EHRs to patients and their number is set to grow.^10 11^ By many accounts, the practice has increased patients’ engagement in care^12^ and enhanced their health understanding,^13^ particularly among marginalised patient groups.^14^ Alongside these benefits, however, new challenges have emerged, including the increasing report of errors in the EHR, or so-called EHRrors.

Patients and HCPs have found inaccuracies in health records before EHRs too. Early studies from the UK found that when patients in primary care were given carbon copies of their record, 1 in 10 noticed an error.^15^ This, however, is not an EHRror.

### What is an EHRror?

We define EHRror as any information about the patient and their care that is documented in the EHR incorrectly or that should be documented but is missing. The term does not describe medical errors that take place in the clinical practice, for example, administering the wrong medication. Instead, EHRrors capture the incongruency between information that is perceived as factually accurate by patients or HCPs and its documentation in the EHR. Initial definitions of an EHRror can be found in box 1.

Box 1 **What does error stand for in EHRror?**

An EHRror could be

- **Error of commission**: information present in the record that is partially or fully incorrect; *example: incorrect allergy listed*.
- **Error of omission**: information that is clinically relevant and should be in the record but is missing; *example: missing information about a serious allergic reaction*.

An EHRror could not be

- **Clinical judgement error**: mistakes made in the interpretation of symptoms, selection of treatment or decision of diagnosis; *example: symptom interpreted as acid reflux instead of a heart attack*.
- **System error**: error related to the software or hardware that host the record; *example: 404 ‘Not found’ error.*
  EHRror, electronic health record error.

EHRrors can be introduced into the EHR by an HCP who populates the electronic medical record (EMR), a patient who has the ability to add their health information through a patient-accessible EHR (PAEHR) or an automated system that imports the information from an external source. While EHR, EMR and PAEHR have distinct definitions (see box 2), they are commonly used interchangeably.^16^ For this reason, we accept EHRrors to mean an error embedded within any of these systems.

Box 2 **What does EHR stand for in EHRror?**

- **EMR**: an electronic record of health-related information about a patient that can be created, gathered, managed and consulted by authorised clinicians and staff within a healthcare organisation.^35^
- **EHR**: records that contain historical data about a patient that are compiled from all local EMRs.^36^
- **PAEHR**: a digital service that provides patients secure online access to their EHRs made available by their healthcare provider.^37^
- **Open notes**: visit note summaries, or the narrative, free-text entries, written by clinicians about a patient, and accessible by the patient online. Open notes constitute a part of a PAEHR.^38^

EHR, electronic health record; EMR, electronic medical record; EHRror, electronic health record error; PAEHR, patient-accessible EHR.

Reflecting the increase in the number of studies on EHRs, research on EHRrors is also growing. Some estimate that as many as every other patient has identified a serious EHRror,^17^ and over half of HCPs expect the majority of patients to find a serious EHRror.^18^ What kind of EHRrors, what impact they have and how widespread they are remains challenging to estimate due to the lack of systematic investigation. Considering the evolving role of the EHR, understanding the impact of EHRrors on its users is crucial.

### Objectives

The aim of this review is to map the evidence on the types and impacts of EHRrors as encountered by patients and HCPs. The review will not address errors that arise during the use of EHR data for secondary purposes, for example, research. To comprehensively explore EHRrors, the systematic review will have the following objectives:

- To determine the frequency with which patients and HCPs encounter EHRrors.
- To identify the types of EHRrors reported by patients and HCPs.
- To assess the impact EHRrors have on patients and HCPs.

## Methods and analysis

To address the objectives, we will carry out a mixed systematic review. The systematic approach was chosen in place of meta-analytical or scoping review for several reasons. First, the vast majority of studies in this area are observational. Conducting a meta-analysis solely on observational data may yield unreliable results due to publication bias; therefore, systematic reviews are generally recommended as a more appropriate approach.^19^ A preliminary literature review further revealed significant variability in how the frequency of EHRrors is reported. With few studies providing quantitative data, these inconsistencies make a meta-analysis unfeasible. While a scoping review could provide a broad overview of the topic, it is not designed to generate detailed insights or answer the clinically relevant and specific research questions (RQs) required for this review.^20^ We therefore chose to carry out a mixed systematic review to best analyse the published evidence on EHRrors.

The protocol for the review has been registered on the International Prospective Register for Systematic Reviews (PROSPERO) database: CRD42024622849. The protocol was developed following the Preferred Reporting Items for Systematic Review and Meta-analysis Protocols (PRISMA-P) guideline and is reported following the PRISMA-P checklist (see online supplemental appendix A).^21^ Research on the project began in October 2024. The review will commence on final peer-review approval of the protocol, and findings are expected to be released at the end of 2025. If substantial amendments to the protocol are necessary, an update to the protocol will be registered in PROSPERO. In such a case, a table listing the changes alongside rationale will be added to the full review publication as per best practice guidelines.^22^

### Research questions

Based on the objectives, three RQs were formulated (see box 3).

Box 3 **Research questions**

- **RQ 1**. How frequently do patient and HCP users of EHRs identify information in the EHR as incorrect or critically omitted?
- **RQ 2**. What types of EHR information are identified as incorrect or critically omitted by patient and HCP users of EHRs?
- **RQ 3**. What impact does the patient-identified or HCP-identified incorrect or critically omitted EHR information have on patients and HCPs?
  EHR, electronic health record; HCP, healthcare professional.

### Eligibility criteria

Study selection criteria based on the Population–Exposure–Outcome framework are summarised in table 1.

**Table 1:** PEO statement

Inclusion criteria	Exclusion criteria
**Population**	
Patients and HCPs who have used EHRs	Patients and HCPs who have not used EHRs
Patients who are proxy and shared access EHR users, such as guardians, parents, informal caregivers or care partners	
**Exposure**	
EHRs implemented at a national, regional or institutional level	Paper-based health records
EHRs accessible online by patients and HCPs	EHRs accessible to patients or HCPs through other methods, for example, a carbon copy
Tethered PHRs	Untethered PHRs
**Outcome**	
**Primary**: patient-reported or HCP-reported EHRrors’ prevalence and descriptions	EHRrors identified through an ML algorithm
**Secondary**: patient or HCP descriptions of EHRror impact on them or on care.	

EHR, electronic health record; EHRror, electronic health record error; HCP, healthcare professional; ML, machine learning; PEO, Population–Exposure–Outcome; PHR, personal health records.

#### Study design

We will include publications that present original research data gathered through (1) observational studies, such as cross-sectional studies, case reports and cohort studies; (2) randomised-control trials, including pilot trials, feasibility studies and cohort studies; (3) quasi-experimental studies, such as non-randomised control trials; and (4) mixed and qualitative studies, such as focus groups, interviews and Delphi studies. Conference papers that have undergone peer review will be considered, given that they present sufficient details on methodology, analysis and results. Publications that do not present original research data will be excluded. These are typically commentaries, letters, editorials, protocols, opinion or statement papers, reviews or framework and methodology papers. Exceptions will be made for cases where, for example, a letter includes original data accompanied by sufficient methodological and analytical details.

#### Setting and timing

The review aims to explore the rate and type of errors found in deployed EHRs as opposed to prototypes or digital solutions developed for research purposes only. For this reason, we will only include studies that report on patient and HCP users’ experiences with EHR systems used as part of healthcare. There will be no restrictions based on timing such as length of exposure to EHRs or time of outcomes collection.

#### Publication characteristics

##### Publication timeframe

**Figure 1:**
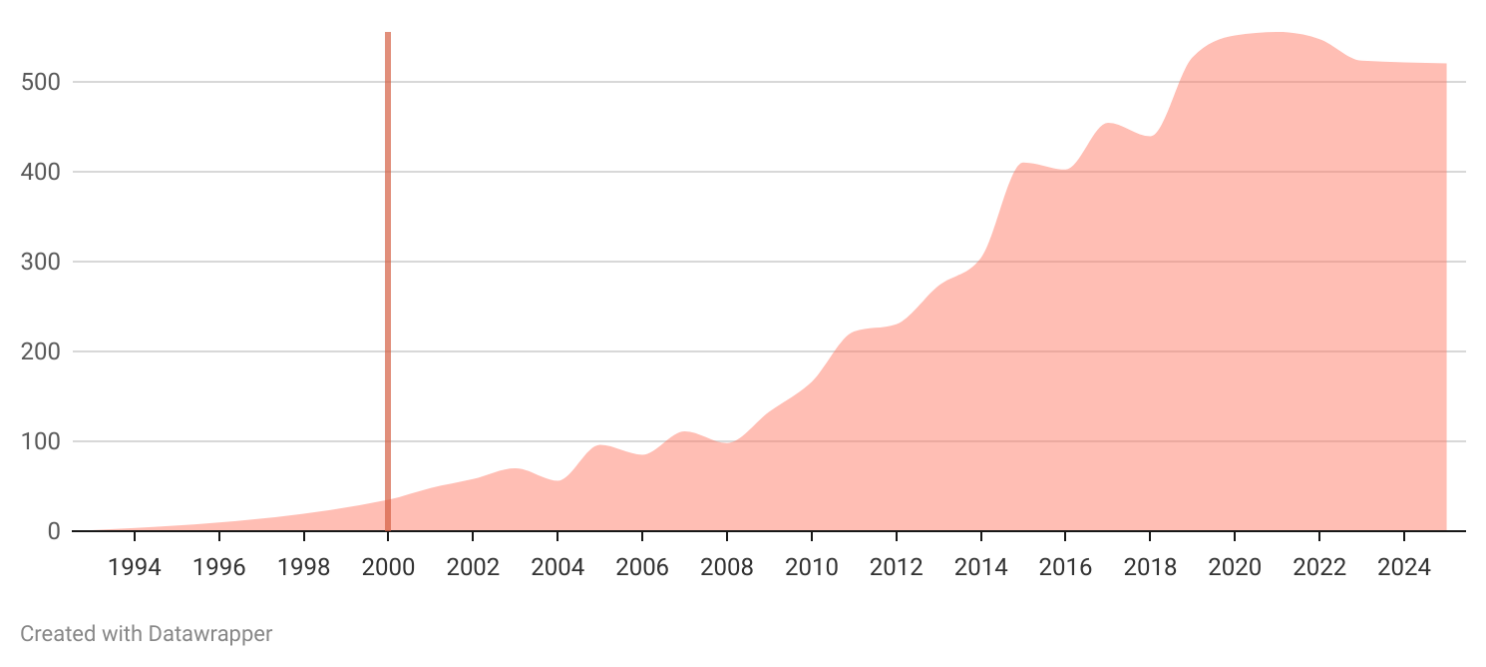
Number of annually published studies identified through a preliminary search in PubMed.

Using the search string (see section ‘Search strategy’), a preliminary literature search found the earliest relevant study from 1993, with a gradual increase in publications until 2008, followed by an exponential growth in the number of studies thereafter (see figure 1). The growing publication rate around the turn of the millennium is expected as some of the first EHR systems began being widely adopted, for example, the 1990s in the USA and 2000s in the EU.^23 24^ To focus on experiences with healthcare-integrated EHRs that are accessible both by patients and HCPs, we will include publications from 2000 onwards.

##### Language

The search itself will be conducted in English, so titles and abstracts must be available in English to be captured. During full-text screening, we will include studies reported in the languages spoken by the research team. At present, these include English, Finnish, German, Hindi, Norwegian, Swedish and Russian.

##### Publication status

Studies published in a journal or conference proceedings following single-blinded and double-blinded peer-review, as well as preprints, will be considered. We will exclude government, technical and non-government organisation reports, theses and dissertations, posters and conference abstracts, clinical trials posted in trial registries, policy briefs, white papers, book chapters and materials accompanying data sets on data repositories.

### Information sources

We will search PubMed (PubMed interface), Web of Science (Web of Science interface) and CINAHL (EBSCO interface) and will not search trial registries and databases. Additional publications may be identified through a manual search of the reference lists in the included publications or similar reviews. If the full text of a potentially eligible report is not available, the publication’s corresponding author will be contacted.

### Search strategy

The search strategy was developed through an iterative process by AK and librarian Görel Sundström (GS) at Uppsala University Library. The search string was reviewed independently by a second librarian, Malin Barkelind, at Uppsala University Library. Seed references were used to compile the initial list of search terms, with further refinement through discussions between the author AK and GS. The review terms along the possible search string terms are summarised in table 2, and an example search string can be found in online supplemental appendix B. The search string will be adapted to the syntax of each database and reported following the PRISMA-S checklist in the full review.^25^

**Table 2:** Review and search terms for search string

Review term	Possible search terms
EHR	Electronic Health Records [MeSH], Patient Portals [MeSH], Health Records, Personal [MeSH:NoExp]
	ambulatory note*, appointment note*, clinic* note*, computeri*ed medical record*, doctor* note*, e record*, electronic record*, EHR, EMR, Electronic medical record*, electronic report*, health electronic record*, health* record*, inpatient portal*, lab result*, laboratory result*, mental health note*, open notes, opennotes, PAEHR, PEHR, patient* portal*, patient* record*, patient web portal*, progress note*, psychiatric note*, psychotherap* note*, test result*, therapy note*, visit* note*, discharge note*, consultation note*, patient history, physical exam*, imaging note*, laboratory test*, pathology note*, procedure note*, narrative report*
EHRror	Medical Errors [MeSH], Data Accuracy [MeSH]
	accura* communication, accura* documentation, accurra* information, accura* record, correct*, error*, failure, false, inaccurate, inaccuracy, incomplete, incorrect*, missing, wrong, accura*
	deviation, misconception, misinterpretation, misspell*, mistake*, misunderstanding, mix up, omission*, oversight*, improp*, inappropriate*, unsuitab*
Frequency and type	amount*, count*, frequenc*, incidence*, instance*, kind*, number*, prevalence, quality, rate, type
Patient and HCP	Patient Access to Records [MeSH], Health Personnel [MeSH], Patients [MeSH]
	inpatient*, patient*, outpatient*, out-patient*, caregiver*, care giver*, care provider*, caretaker*, care taker*, carer, care partner, clinician*, doctor*, general practitioner*, GP, healthcare professional*, health care professional*, HCP*, healthcare provider*, medical secretar*, nurse*, physician*, therapist*, psychotherapist*, psychologist*, resident*

EHR, electronic health record; EHRror, electronic health record error; HCP, healthcare professional.

The search will be conducted at the beginning of the review and repeated near its conclusion to ensure the inclusion of the most recent publications. The PROSPERO database will also be periodically searched for ongoing or recently completed reviews that might highlight relevant studies. Any such studies identified will be manually incorporated into the review.

### Records management

#### Data management

Once collected, search results will undergo de-duplication in EndNote. This will be undertaken by librarian GS. The de-duplicated search results will then be transferred to AK, who will upload them to Covidence, where the screening process will take place. It is likely that some duplicate entries will persist and some studies will have their results across multiple publications. To avoid double-counting data, reviewers will be trained to identify such instances and address them appropriately, such as by manually removing true duplicates.

#### Selection process

We will adopt a biphasic selection process. First, two reviewers will independently screen the title and abstract. No blinding will be applied to the author names, author affiliations or journal. Reviewers will be guided by a decision tree that contains key terminology and inclusion criteria. Publications that pass title and abstract screening will proceed to full-text screening. There, a pair of reviewers will further independently verify if the study meets the selection criteria and presents sufficient information on the outcomes of interest. Any disagreements during both phases will be arbitrated by a third reviewer (authors AK or MH), with justification for the decision communicated to the original review pair. Regular calibration meetings will be held to ensure clarity on the inclusion criteria.

#### Data collection process

Studies that passed both phases of the screening will proceed to extraction. There, reviewers will work in pairs to extract data in a preset extraction template. One reviewer in a pair will initially populate the template, and the other will verify it for accuracy and completion. Disagreements will be resolved in the first instance through a discussion within the pair, and, if necessary, further involve a third reviewer for a final decision (AK). Publication authors may be contacted if further clarifications about the presented methodology or data are needed.

#### Data items

For each study, we will extract data in the following categories:

- *Publication details*: year of publication, authors and pre-registration.
- *Setting*: country and EHR platform.
- *Participant characteristics*: type of participant (eg, patient or HCP), patient characteristics (eg, health conditions), HCP characteristics (eg, specialty), gender and age.
- *EHRror prevalence*: rate within and across participants and number of errors.
- *EHRror type*: descriptions of EHRror and EHRror category (eg, error of omission).
- *EHRror impact*: perceived seriousness by patient or HCP, descriptions of impact on patient, HCP or care and severity of impact.

Data will be extracted directly from the text or approximated from figures. Qualitative data will either be copied verbatim or, if excessively voluminous, summarised and abstracted. Abstraction will be conducted by reviewers experienced in qualitative data analysis, aiming to strike a balance between preserving detailed information and capturing overarching narrative trends.

### Outcomes and prioritisation

Primary outcomes will be the prevalence of EHRrors identified by patients and HCPs, and the description of EHRrors. Prevalence is expected to be expressed through quantitative data such as a percentage of surveyed patients who report to have found an EHRror. Surrogate outcomes will also be accepted due to the small number of quantitative studies that we predict to have consistently investigated EHRrors. These could include qualitative descriptions, for example, ‘some participants’. Descriptions of EHRrors are expected to range from short phrasal descriptors to long explanations, reflecting the study methodology. They will be summarised as EHRror types and listed under the broader EHRror categories (see box 2). Depending on data saturation, a composite outcome of frequency of EHRrors per type will also be reported. Secondary outcome will be descriptions of any impact EHRrors have had on patients and HCPs. To enhance the clinical relevance of the review, EHRrors impact will be described referring to established EHRror types and the context of participant characteristics.

### Data synthesis

Quantitative and qualitative evidence synthesis will be carried out following the Joanna Briggs Institute Framework for Mixed Systematic Reviews.^26^ We will apply the convergent integrated approach, in which quantitative synthesis is integrated with qualitative to complement evidence and gain higher depth of insight.^27^ First, we will aggregate the quantitative data on EHRrors prevalence. Due to the high variability in how EHRrors prevalence is quantified in the literature, we will not carry out statistical pooling but instead create tabular and narrative summaries. Then, we will aggregate qualitative data into higher-order statements, grouped through semantic themes. Where synergies between quantitative and qualitative data exist, the former will undergo transformation through qualitisation. Lastly, aiming to address each RQ, the quantitative and qualitative syntheses will be integrated and organised into research findings.

### Critical appraisal

#### Meta-biases and risk of bias in individual studies

The reviewed studies may include meta-biases pertaining to publication or selective outcome reporting. To evaluate this, each included publication will be assessed against its protocol, pre-registration or preprint, if available. Non-randomised studies are particularly vulnerable to meta-biases, yet the registration of planned analyses is less common compared with randomised trials. To mitigate this limitation, preprints will be included in the search strategy to capture relevant data and improve transparency.

To assess the risk of bias in mixed-methods studies, we will apply the Mixed Methods Appraisal Tool (MMAT).^28^ The MMAT was chosen as it will enable consistent appraisal across a variety of study designs. Two reviewers will complete the MMAT for each study independently. Where scoring deviations occur, a consensus meeting will be carried out to resolve them. If a reviewer is an author of an assessed study, another reviewer will be assigned to prevent bias. Results of the MMAT will be published as a supplementary file.

#### Confidence in cumulative evidence

Next, we will appraise the confidence in the generated cumulative evidence using the Confidence in the Evidence from Reviews of Qualitative Research (GRADE-CERQual) framework.^29^ To do this, two reviewers will independently prepare a CERQual qualitative evidence profile for each review finding, grouping them by RQ. The CERQual profile includes assessment of methodological limitations, relevance, coherence and adequacy of the evidence supporting a review finding.^30^ Each finding will receive an overall confidence rating. Disagreements in ratings will be resolved through consensus meetings. To promote transparency, the CERQual evidence profiles will be created using the interactive Summary of Qualitative Findings (iSoQ) tool by GRADE and stored in the iSoQ open-access database. A Summary of Qualitative Findings table will be prepared for the main text of the review summarising the appraisal.

#### Reflexivity statement

The authors form an international multidisciplinary team whose professional and personal expertise will shape the interpretation of the systematic review findings. Following the AMEE Guide on reflexivity,^31^ the authors considered how their subjectivity and context would shape their contribution to the project. AK, a healthcare researcher studying patient and HCPs’ perspectives on EHRrors, will provide expertise on EHRrors. MH, an implementation scientist, will contribute with in-depth knowledge on PAEHR implementation and use in different socio-technical contexts, as well as personal experience of finding EHRrors. DA is a researcher in obstetrics and gynaecology and will contribute with knowledge of maternal healthcare. TSD is a researcher within health informatics, with a focus on patient contribution in healthcare regarding the use of digital tools. JHa is a PhD student in health informatics knowledgeable about EHRrors in the context of proxy access and young patients. KH is a researcher in chronic pain and will use her knowledge of healthcare related to chronic pain patients. JHu is a patient researcher with interest in co-production of healthcare, patient knowledge and lived experience of harms associated with EHRrors. YTL is a senior physician and specialist in Internal Medicine and will contribute with clinical experience from working with EHRs and handling EHRrors in the clinical setting. CL and SAR carry out meta-analysis research on chronic pain interventions and will assist with the conduct of the review to a high standard. A-JN is a medical doctor who brings clinical experience alongside a global health perspective for an inclusive interpretation of the review findings. BM is a general practitioner (family doctor) and health psychologist, and will bring his experience of writing entries in patients’ EHRs during his clinical work. JS, a psychiatrist, will contribute clinical and research experience on PAEHR and EHRrors in psychiatry and psychotherapy. SS researches eHealth services’ usability and accessibility, and has first-hand account of parental proxy access and secondhand of caregiver proxy for a person with disability. CB, a health informaticist and medical philosopher, brings professional expertise and personal insight, having experienced errors in her own records. The reflexivity statement will be revisited and updated as needed in the review publication.

### Presentation of findings

**Figure 2:**
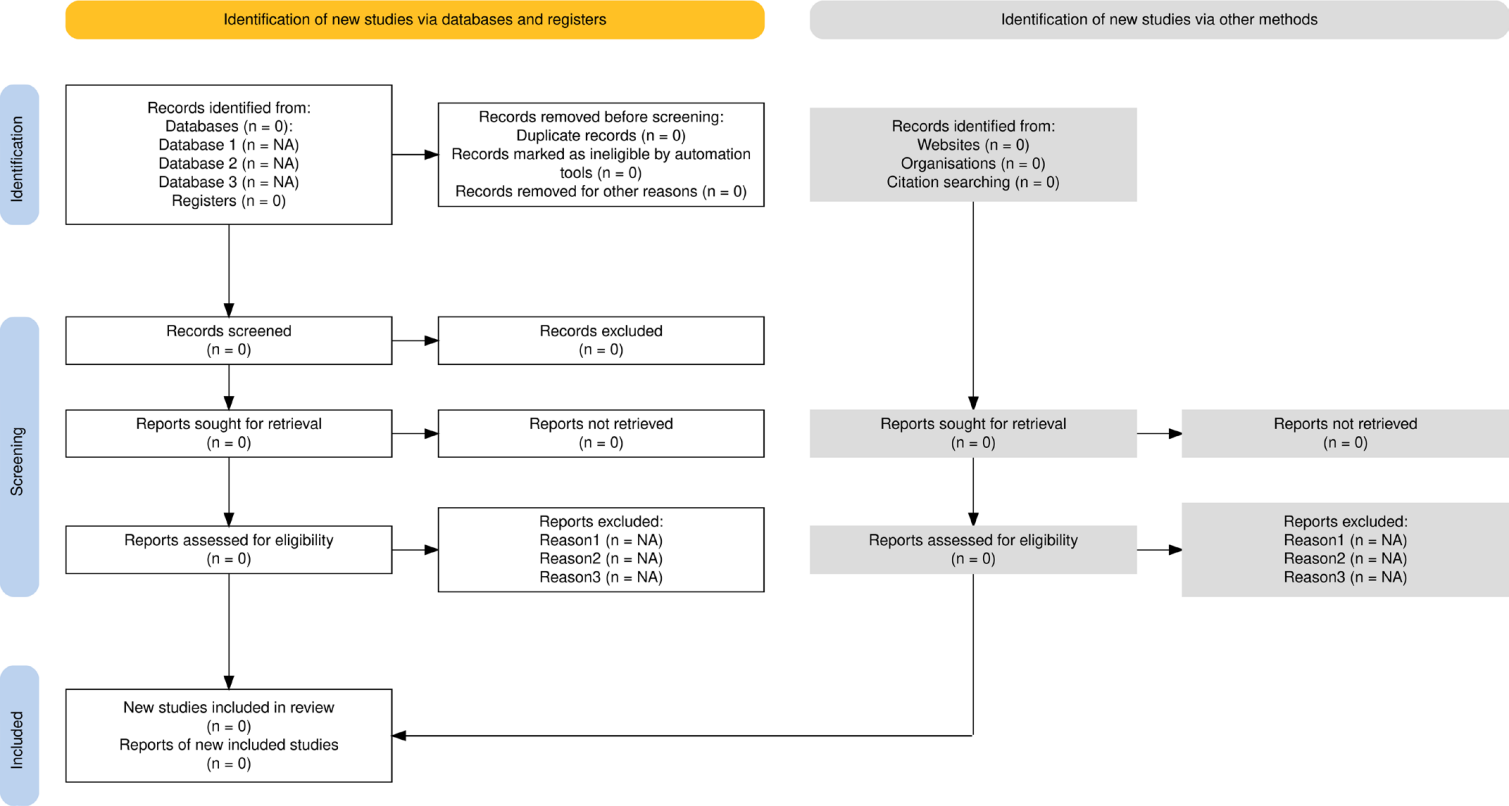
Preferred Reporting Items for Systematic Reviews and Meta-Analyses 2020 flow diagram template that will be updated for the full review. Adapted from Haddaway *et al*.^33^

The results of the systematic review will be reported following the PRISMA 2020 Checklist to minimise risk of bias.^32^ A PRISMA 2020 flow diagram will be generated to illustrate the screening process (see figure 2).^33^ Results will comprise tabular and narrative syntheses of quantitative and qualitative data. Findings will be interpreted in close cooperation with patient representatives and HCPs.

## Discussion

This protocol outlines a mixed systematic review designed to address critical gaps in the understanding of EHRrors, including their prevalence, types and impacts on patients and HCPs. By synthesising quantitative and qualitative data, this review aims to provide a comprehensive and nuanced understanding of EHRrors, an area that remains underexplored despite its significant implications for patient safety and healthcare delivery.^34^

The review will be the first to systematically examine EHRrors identified by both patients and HCPs, offering insights into a growing yet fragmented body of literature. By integrating findings from diverse study designs, this review will provide guidance on what constitutes an EHRror, the frequency of their occurrence and their effects on clinical care. These findings will not only enrich academic discourse but also inform practical strategies for improving EHR systems and training programmes for healthcare providers.

A key strength of this review will be its methodological rigour, adhering to best practices when it comes to transparent and reproducible reporting, as well as the critical appraisal of the confidence in cumulative findings. This approach will promote the further use of the review findings in clinical recommendations. The inclusion of patient representatives and HCPs in interpreting and contextualising the results adds further validity and relevance to the study.

The review, however, will have some limitations. As the search will be conducted in English, only studies with English titles and abstracts will be screened, which may exclude relevant research published in other languages. To partially mitigate this, full texts in languages spoken by the research team will be included where applicable. Another limitation is the variability in how EHRrors are reported across studies, which may complicate the synthesis of quantitative data. Additionally, the inclusion of preprints and non-randomised studies, while necessary to capture the breadth of the evidence, introduces a potential risk of bias. Finally, although secondary use of EHR data lies outside the scope of this review, the presence of EHRrors represents a fundamental obstacle to data reliability, particularly to EHR-based research. The findings may therefore have indirect implications for strengthening the quality of EHR data used in research but this will not be evaluated. To tackle these challenges, an update to the review will be planned in due course.

### Implications for research, practice and policy

The findings of this review will have significant implications for research, clinical practice and policymaking. A deeper understanding of EHRrors can guide the development of more robust and user-friendly EHR systems, improve patient safety protocols and support evidence-based policymaking around EHR implementation and maintenance. The results may also encourage further research into mitigating the occurrence and impact of EHRrors and foster collaboration between patients, healthcare providers and system developers. In addition, by identifying patterns in how EHRrors are currently defined and reported, the review may inform future efforts to promote greater consistency in their documentation and measurement in research and practice.

In conclusion, this mixed systematic review represents an important step towards addressing the pervasive yet underappreciated issue of EHRrors. By illuminating the experiences of both patients and HCPs, the review aims to contribute to safer and more effective healthcare documentation systems worldwide.

## Ethics and dissemination

The study will not involve collection or analysis of individual patient data, so no ethical approval will be sought. Results will be published in a peer-reviewed publication and further disseminated through scientific events and educational materials.

## Supplementary material

10.1136/bmjopen-2024-098241 online supplemental file 1

10.1136/bmjopen-2024-098241 online supplemental file 2
