# Supplementary material for: Prevalence and types of errors in the electronic health record: protocol for a mixed systematic review
Source: BMJ Open. 2025 Jun 9;15(6):e098241. doi: 10.1136/bmjopen-2024-098241 (PMC12161391; doi:10.1136/bmjopen-2024-098241)
Supplement: online supplemental file 2 [file bmjopen-15-6-s002.docx]

**Appendix B.** Example search string for PubMed

(("Electronic Health Records"[MeSH Terms] OR "Patient Portals"[MeSH Terms] OR "health records, personal"[MeSH Terms:noexp] OR ("ambulatory note*"[Title/Abstract] OR "appointment note*"[Title/Abstract] OR "clinic* note*"[Title/Abstract] OR "computeri*ed medical record*"[Title/Abstract] OR "consultation note*"[Title/Abstract] OR "discharge note*"[Title/Abstract] OR "doctor* note*"[Title/Abstract] OR "e record*"[Title/Abstract] OR "electronic record*"[Title/Abstract] OR "EHR"[Title/Abstract] OR "EMR"[Title/Abstract] OR "electronic medical record*"[Title/Abstract] OR "electronic report*"[Title/Abstract] OR "health electronic record*"[Title/Abstract] OR "health* record*"[Title/Abstract] OR "imaging note*"[Title/Abstract] OR "inpatient portal*"[Title/Abstract] OR "mental health note*"[Title/Abstract] OR "narrative report*"[Title/Abstract] OR "open notes"[Title/Abstract] OR "opennotes"[Title/Abstract] OR "PAEHR"[Title/Abstract] OR "PEHR"[Title/Abstract] OR "pathology note*"[Title/Abstract] OR "patient history"[Title/Abstract] OR "patient* portal*"[Title/Abstract] OR "patient* record*"[Title/Abstract] OR "patient web portal*"[Title/Abstract] OR "progress note*"[Title/Abstract] OR "psychiatric note*"[Title/Abstract] OR "physical exam*"[Title/Abstract] OR "procedure note*"[Title/Abstract] OR "psychotherap* note*"[Title/Abstract] OR "test result*"[Title/Abstract] OR "therapy note*"[Title/Abstract] OR "visit* note*"[Title/Abstract])) AND ("Medical Errors"[MeSH Terms] OR "Data Accuracy"[MeSH Terms] OR ("Accurate communication"[Title/Abstract:~5] OR "Accuracy communication"[Title/Abstract:~5] OR "correct communication"[Title/Abstract:~5] OR "correctly communication"[Title/Abstract:~5] OR "error communication"[Title/Abstract:~5] OR "errors communication"[Title/Abstract:~5] OR "failure communication"[Title/Abstract:~5] OR "false communication"[Title/Abstract:~5] OR "inaccurate communication"[Title/Abstract:~5] OR "inaccuracy communication"[Title/Abstract:~5] OR "incomplete communication"[Title/Abstract:~5] OR "incorrect communication"[Title/Abstract:~5] OR "incorrectly communication"[Title/Abstract:~5] OR "missing communication"[Title/Abstract:~5] OR "wrong communication"[Title/Abstract:~5] OR "Accurate documentation"[Title/Abstract:~5] OR "Accuracy documentation"[Title/Abstract:~5] OR "correct documentation"[Title/Abstract:~5] OR "correctly documentation"[Title/Abstract:~5] OR "error documentation"[Title/Abstract:~5] OR "errors documentation"[Title/Abstract:~5] OR "failure documentation"[Title/Abstract:~5] OR "false documentation"[Title/Abstract:~5] OR "inaccurate documentation"[Title/Abstract:~5] OR "inaccuracy documentation"[Title/Abstract:~5] OR "incomplete documentation"[Title/Abstract:~5] OR "incorrect documentation"[Title/Abstract:~5] OR "incorrectly documentation"[Title/Abstract:~5] OR "missing documentation"[Title/Abstract:~5] OR "wrong documentation"[Title/Abstract:~5] OR "Accurate information"[Title/Abstract:~5] OR "Accuracy information"[Title/Abstract:~5] OR "correct information"[Title/Abstract:~5] OR "correctly information"[Title/Abstract:~5] OR "error information"[Title/Abstract:~5] OR "errors information"[Title/Abstract:~5] OR "failure information"[Title/Abstract:~5] OR "false information"[Title/Abstract:~5] OR "inaccurate information"[Title/Abstract:~5] OR "inaccuracy information"[Title/Abstract:~5] OR "incomplete information"[Title/Abstract:~5] OR "incorrect information"[Title/Abstract:~5] OR "incorrectly information"[Title/Abstract:~5] OR "missing information"[Title/Abstract:~5] OR "wrong information"[Title/Abstract:~5] OR "Accurate record"[Title/Abstract:~5] OR "Accuracy record"[Title/Abstract:~5] OR "correct record"[Title/Abstract:~5] OR "correctly record"[Title/Abstract:~5] OR "error record"[Title/Abstract:~5] OR "errors record"[Title/Abstract:~5] OR "failure record"[Title/Abstract:~5] OR "false record"[Title/Abstract:~5] OR "inaccurate record"[Title/Abstract:~5] OR "inaccuracy record"[Title/Abstract:~5] OR "incomplete record"[Title/Abstract:~5] OR "incorrect record"[Title/Abstract:~5] OR "incorrectly record"[Title/Abstract:~5] OR "missing record"[Title/Abstract:~5] OR "wrong record"[Title/Abstract:~5] OR "Accurate records"[Title/Abstract:~5] OR "Accuracy records"[Title/Abstract:~5] OR "correct records"[Title/Abstract:~5] OR "correctly records"[Title/Abstract:~5] OR "error records"[Title/Abstract:~5] OR "errors records"[Title/Abstract:~5] OR "failure records"[Title/Abstract:~5] OR "false records"[Title/Abstract:~5] OR "inaccurate records"[Title/Abstract:~5] OR "inaccuracy records"[Title/Abstract:~5] OR "incomplete records"[Title/Abstract:~5] OR "incorrect records"[Title/Abstract:~5] OR "incorrectly records"[Title/Abstract:~5] OR "missing records"[Title/Abstract:~5] OR "wrong records"[Title/Abstract:~5] OR ("deviation*"[Title/Abstract] OR "misconception*"[Title/Abstract] OR "misinterpretation*"[Title/Abstract] OR "misspell*"[Title/Abstract] OR "mistake*"[Title/Abstract] OR "Misunderstanding"[Title/Abstract] OR "mix up"[Title/Abstract] OR "omission*"[Title/Abstract] OR "omitted"[Title/Abstract] OR "oversight*"[Title/Abstract] OR "deprec*"[Title/Abstract] OR "improp*"[Title/Abstract] OR "inappropriate*"[Title/Abstract] OR "unsuitab*"[Title/Abstract]))) AND ("amount*"[Title/Abstract] OR "count*"[Title/Abstract] OR "event*"[Title/Abstract] OR "frequen*"[Title/Abstract] OR "incidence*"[Title/Abstract] OR "instance*"[Title/Abstract] OR "Kind"[Title/Abstract] OR "kinds"[Title/Abstract] OR "number*"[Title/Abstract] OR "Prevalence"[Title/Abstract] OR "rate"[Title/Abstract] OR "type*"[Title/Abstract] OR "perceive*"[Title/Abstract] OR "record review"[Title/Abstract]) AND ("Patient Access to Records"[MeSH Terms] OR "Health Personnel"[MeSH Terms] OR "Patients"[MeSH Terms] OR ("inpatient*"[Title/Abstract] OR "patient*"[Title/Abstract] OR "out patient*"[Title/Abstract] OR "outpatient*"[Title/Abstract] OR "guardian*"[Title/Abstract] OR "parent*"[Title/Abstract] OR "administrator*"[Title/Abstract] OR "attending"[Title/Abstract] OR "care giver*"[Title/Abstract] OR "care provider"[Title/Abstract] OR "care taker*"[Title/Abstract] OR "caregiver*"[Title/Abstract] OR "Carer"[Title/Abstract] OR "caretaker*"[Title/Abstract] OR "clinician*"[Title/Abstract] OR "doctor*"[Title/Abstract] OR "general practitioner*"[Title/Abstract] OR "HCP"[Title/Abstract] OR "health care professional*"[Title/Abstract] OR "health care provider*"[Title/Abstract] OR "healthcare professional*"[Title/Abstract] OR "healthcare provider*"[Title/Abstract] OR "hospitalist"[Title/Abstract] OR "medical secretar*"[Title/Abstract] OR "nurse*"[Title/Abstract] OR "physician*"[Title/Abstract] OR "Therapist"[Title/Abstract] OR "Psychotherapist"[Title/Abstract] OR "Psychologist"[Title/Abstract] OR "resident"[Title/Abstract]))) AND (2000:2025[pdat])
