# Supplementary material for: Prevalence and types of errors in the electronic health record: protocol for a mixed systematic review
Source: BMJ Open. 2025 Jun 9;15(6):e098241. doi: 10.1136/bmjopen-2024-098241 (PMC12161391; doi:10.1136/bmjopen-2024-098241)
Supplement: Supplementary file 3 [file bmjopen-2024-098241.reviewer_comments.pdf]

PEER REVIEW HISTORY

BMJ Open publishes all reviews undertaken for accepted manuscripts. Reviewers are asked to complete a checklist review form and are provided with free text boxes to elaborate on their assessment. These free text comments are reproduced below.

ARTICLE DETAILS

Title (Provisional)

Prevalence and types of errors in the electronic health record: Protocol for a mixed systematic review

Authors

Kharko, Anna; Hagglund, Maria; Angelova, Dafina; Scott Duncan, Therese; Hagström , Josefin; Hansford, Kirralise; Hunt , Joanne; Trolle Lagerros, Ylva; Locher, Cosima; McMillan, Brian; Nivins , Asha-Judy; Rosch, Sophie Anna; Schwarz, Julian; Simola, Saija; Blease, Charlotte R

VERSION 1 - REVIEW

Reviewer 1
Name Ricotta, Emily
Affiliation Uniformed Services University of the Health Sciences, Preventive Medicine and Biostatistics
Date 07-Mar-2025
COI None

Thank you for the opportunity to review "Prevalence and types of errors in the electronic health record: Protocol for a mixed systematic review."

This protocol is very well written (although it took me a while to stop laughing at EHRrors because I kept reading it like a pirate). The completed review will be an important contribution to the literature. I have only minor comments:

1. You could make a stronger point earlier in your protocol that your review is NOT assessing errors impacting research using EHRs. Upon first reading, I thought "you aren't collecting data from EHR researchers, so you will miss valuable errors." Then it became clearer that you are really focused on errors at the point of data capture and the impact on patient care. Obviously correcting the errors on which you are focused will improve research downstream, but those are not the data you are capturing in your review.

2. In your EHRror definition, do you include typos and duplications? How will you handle data errors where missingness is unknown to the HCP because the patient saw an out of network provider (or is this only a problem in the US?)

3. Are you planning to quantify seriousness of the errors? E.g., a typo < omission < incorrect diagnosis.

4. I recommend restructuring Table 1 to better agree with the "outcomes and prioritisation" section, specifically highlighting primary and secondary outcomes.

5. Did you consider using the ROBINS-E tool for quantifying bias in non-randomized studies? It's kind of a pain, but if you're going to the trouble of using the MMAT and GRADE-CERQual, it seems like an omission to not include the ROBINS-E.
https://www.riskofbias.info/welcome/robins-e-tool

6. Discussion, line 378 - you say a limitation is that you are only including English language studies, but earlier in the protocol you mention including all languages of study team. Please harmonize language.

7. Since you mention the "high variability in how EHRrors prevalence is quantified in the literature," I would love to see a discussion somewhere in your review on recommendations to standardize recording and reporting of EHRrors.

**Reviewer**	**2**
**Name**	**Trerotoli, Paolo**
**Affiliation**	**University of Bari Aldo Moro, Interdisciplinary Medicine**
**Date**	**25-Apr-2025**
**COI**	**None**

I appreciate the opportunity to revise this paper that is a study protocol.

The authors used correctly the methodology to avoid biases , as possible.

THe choice of the mixed review doesn't allow to get how they will analyse the results and how they will show, if they will use some statistical method or they will only refer what studies report. A most clear description of this will help to have a more clear description about what the authors want to find and describe, and the possible effect of reaserch that uses EHR.

VERSION 1 - AUTHOR RESPONSE

Reviewer: 1

Dr. Emily Ricotta, Uniformed Services University of the Health Sciences
Comments to the Author:

Thank you for the opportunity to review "Prevalence and types of errors in the electronic health record: Protocol for a mixed systematic review."

This protocol is very well written (although it took me a while to stop laughing at EHRrors because I kept reading it like a pirate). The completed review will be an important contribution to the literature. I have only minor comments:
Response: We thank the reviewer for their thoughtful feedback. Our responses be charted below; set sail with us through each point!

R1.1. You could make a stronger point earlier in your protocol that your review is NOT assessing errors impacting research using EHRs. Upon first reading, I thought "you aren't collecting data from EHR researchers, so you will miss valuable errors." Then it became clearer that you are really focused on errors at the point of data capture and the impact on patient care. Obviously correcting the errors on which you are focused will improve research downstream, but those are not the data you are capturing in your review.
Response: Thank you for this helpful comment. To avoid confusion, we added the following sentence to ***Objectives***: "The review will not address errors that arise during the use of EHR data for secondary purposes, e.g. research." (p. 5)

R1.2. In your EHRror definition, do you include typos and duplications? How will you handle data errors where missingness is unknown to the HCP because the patient saw an out of network provider (or is this only a problem in the US?)
Response: This is a keen observation and one we discussed during protocol development. We decided against defining these as EHRrors at the stage of the protocol, and instead to make a decision during the review. We anticipate that some patient-reported EHRrors will include typographical issues, and that some omissions may arise from contextual factors, for example, like the one you described, or in instances when entries that are hidden from the patient. Rather than impose fixed definitions prematurely, we opted to let the categorisation of these cases be guided by the data patterns that emerge during the review.

R1.3. Are you planning to quantify seriousness of the errors? E.g., a typo < omission < incorrect diagnosis.
Response: We thank the reviewer for raising this point. We do not plan to apply a predefined hierarchy of seriousness but instead, we will be guided by the data. We anticipate that the thematic synthesis will reveal common patterns of perceived severity when patients/HCPs discuss impact, which we will report narratively and, where feasible, descriptively quantify. To make our approach transparent, we have now added *"perceived seriousness by patient or HCP"* to the description of *EHRror impact* in the section ***Data items*** (p. 10).

R1.4. I recommend restructuring Table 1 to better agree with the "outcomes and prioritisation" section, specifically highlighting primary and secondary outcomes.
Response: We have updated Table 1 to more clearly reflect the distinction between primary and secondary outcomes, in alignment with the "Outcomes and prioritisation" section. The primary outcomes (prevalence and types of EHRrors) and the secondary outcome (impact of EHRrors) are now explicitly noted in the Outcome row of the table, while the conventional structure of the PEO table has been retained.

R1.5. Did you consider using the ROBINS-E tool for quantifying bias in non-randomized studies? It's kind of a pain, but if you're going to the trouble of using the MMAT and GRADE-CERQual, it seems like an omission to not include the ROBINS-E.
https://www.riskofbias.info/welcome/robins-e-tool
Response: We considered a range of appraisal tools, including ROBINS-E. However, we found that ROBINS-E is primarily intended for assessing risk of bias in non-randomised studies that estimate the causal effect of an exposure on a health outcome. It is most applicable when exposures are external and measurable — which doesn't fit the subjective, user-reported nature of our “exposure” (i.e., encountering an EHRror). Given the diversity of included study designs (quantitative, qualitative, and mixed-methods), the MMAT was selected as a more appropriate tool.

R1.6. Discussion, line 378 - you say a limitation is that you are only including English language studies, but earlier in the protocol you mention including all languages of study team. Please harmonize language.
Response: We thank the reviewer for catching this inconsistency. We have harmonised the language to clarify that while the search will be performed in English, we will include studies with full texts in languages spoken by the research team (e.g., German, Swedish, Russian) where an English title and abstract are available.

In ***Language***, we now report: *“The search itself will be conducted in English, so titles and abstracts must be available in English to be captured. During full-text screening, we will include studies reported in the languages spoken by the research team”* (p. 7).

In the ***Discussion***: *“The review, however, will have some limitations. As the search will be conducted in English, only studies with English titles and abstracts will be screened, which may exclude relevant research published in other languages. To partially mitigate this, full texts in languages spoken by the research team will be included where applicable. ”* (pp.13-14).

R1.7. Since you mention the "high variability in how EHRrors prevalence is quantified in the literature," I would love to see a discussion somewhere in your review on recommendations to standardize recording and reporting of EHRrors.
Response: We agree, this is an important recommendation we expect to arise from the review. We have added the following to ***Implications for Research, Practice, and Policy***: *“In addition, by identifying patterns in how EHRrors are currently defined and reported, the review may inform future efforts to promote greater consistency in their documentation and measurement in research and practice.”* (p. 14)

Reviewer: 2

Dr. Paolo Trerotoli, University of Bari Aldo Moro

Comments to the Author:

I appreciate the opportunity to revise this paper that is a study protocol.

The authors used correctly the methodology to avoid biases , as possible.

Response: We thank the reviewer for taking the time to provide feedback on our protocol.

R2.1. THe choice of the mixed review doesn't allow to get how they will analyse the results and how they will show, if they will use some statistical method or they will only refer what studies report. A most clear description of this will help to have a more clear description about what the authors want to find and describe, and the possible effect of reaserch that uses EHR.

Response: We appreciate the reviewer's concern about the chosen methodology, but respectfully disagree that this approach will not allow for further analysis. At the start of ***Methods and Analysis***, we stated the reasons for selecting a mixed-methods review instead of a meta-analysis is due to expected high variability in how our outcome data is reported: "*The systematic approach was chosen in place of meta-analytical or scoping review for several reasons. First, the vast majority of studies in this area are observational. Conducting a meta-analysis solely on observational data may yield unreliable results due to publication bias; therefore, systematic reviews are generally recommended as a more appropriate approach. [23] A preliminary literature review further revealed significant variability in how the frequency of EHRrors is reported. With few studies providing quantitative data, these inconsistencies make a meta-analysis unfeasible."* (p. 5)

We also disagree that further elaboration on analytical steps is necessary as a detailed description is already presented in ***Data Synthesis***, e.g. *"Quantitative and qualitative evidence synthesis will be carried out following the Joanna Briggs Institute (JBI) Framework for Mixed Systematic Reviews. [30] We will apply the convergent integrated approach, in which quantitative synthesis is integrated with qualitative to complement evidence and gain higher depth of insight. [31] First, we will aggregate the quantitative data on EHRrors prevalence. Due to the high variability in how EHRrors prevalence is quantified in the literature, we will not carry out statistical pooling but instead create tabular and narrative summaries. Then, we will aggregate qualitative data into higher-order statements, grouped through semantic themes."* (p. 11)

We do, however, agree that a clearer statement about the expected impact of the review on EHR-based research is necessary, so we made the following addition to ***Discussion***: *"Finally, although secondary use of EHR data lies outside the scope of this review, the presence of EHRrors represents a fundamental obstacle to data reliability, particularly to EHR-based research. The findings may therefore have indirect implications for strengthening the quality of EHR data used in research but this will not be evaluated."* (p. 14)
